# Supplementary material for: Efficacy and Safety of Preoperative Melatonin for Women Undergoing Cesarean Section: A Systematic Review and Meta-Analysis of Randomized Placebo-Controlled Trials
Source: Medicina (Kaunas). 2023 Jun 1;59(6):1065. doi: 10.3390/medicina59061065 (PMC10302920; doi:10.3390/medicina59061065)
Supplement: Supplementary file 1 [file medicina-59-01065-s001.zip › Supplemenrary material_19-05-2023.pdf]

Table S1. The details of the search strategy for each database:

PubMed

Strategy: (Melatonin OR pineal hormone OR NSC-113928) AND (Cesarean section OR Caesarean Section OR Abdominal Deliveries OR Abdominal delivery OR C-Section OR C Section OR Postcesarean Section)

Results: 29

Scopus

Strategy: (Melatonin OR “pineal hormone” OR “NSC-113928”) AND (“Cesarean section” OR “Caesarean Section” OR “Abdominal Deliveries” OR “Abdominal delivery” OR “C-Section” OR “C Section” OR “Postcesarean Section”)

Results: 67

Cochrane

Strategy: : (Melatonin OR pineal hormone OR NSC-113928) AND (Cesarean section OR Caesarean Section OR Abdominal Deliveries OR Abdominal delivery OR C-Section OR C Section OR Postcesarean Section)

Results: 26

WOS

Strategy: (Melatonin OR pineal hormone OR NSC-113928) AND (Cesarean section OR Caesarean Section OR Abdominal Deliveries OR Abdominal delivery OR C-Section OR C Section OR Postcesarean Section)

Results: 260

Table S2. Summary of GRADE rating.

Outcome	Risk of bias	Inconsistency	Indirectness	Imprecision	Other considerations [a]	Overall certainty of evidence
Hemoglobin (mg/dl)	Serious [b]	Serious [c]	Not serious	Serious [d]	Not serious	⊕◯◯◯ Very low
Heart rate (bpm)	Serious [b]	Serious [c]	Not serious	Serious [d]	Not serious	⊕◯◯◯ Very low
Mean arterial pressure	Serious [b]	Serious [c]	Not serious	Serious [d]	Not serious	⊕◯◯◯ Very low
Pain score (10-point)	Serious [b]	Serious [c]	Not serious	Serious [d]	Not serious	⊕◯◯◯ Very low
Time to first analgesic request	Serious [b]	Serious [c]	Not serious	Serious [d]	Not serious	⊕◯◯◯ Very low
Total blood loss	Serious [b]	Serious [c]	Not serious	Serious [d]	Not serious	⊕◯◯◯ Very low
Adverse events	Serious [b]	Serious [c]	Not serious	Serious [d]	Not serious	⊕◯◯◯ Very low

[a] Other considerations are publication bias, large efect, dose response, and plausible confounding factors.
[b] As the included studies showed higher risk of bias especially with randomization process besides other bias.
[c] As the outcome had signifcant heterogeneity.
[d] As the analysis included small number of patients with wide confdence interval.
Very low quality— Any estimate of effect is very uncertain.

2 Figures:

Figure S1: Forest plot comparing each dose of the melatonin and the placebo regarding hemoglobin level:

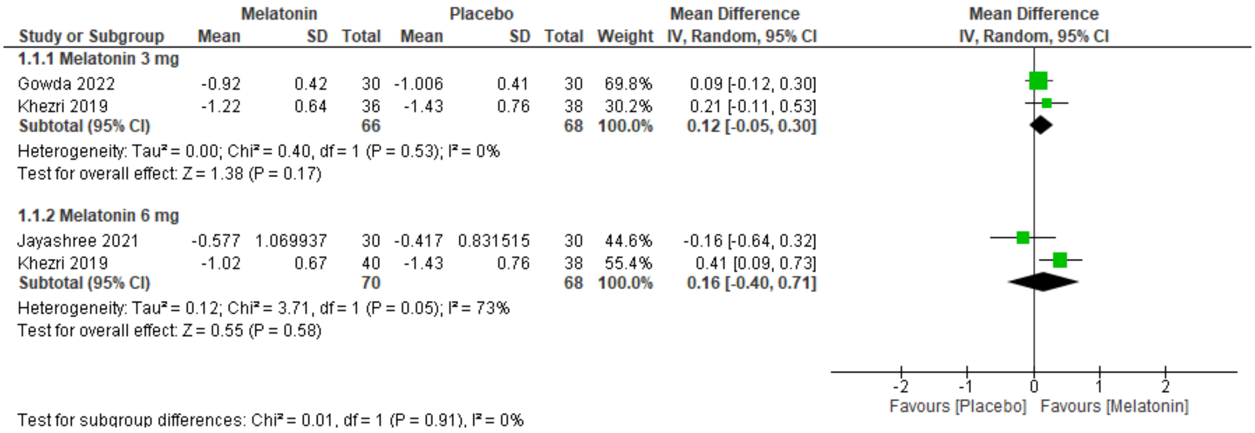

Figure S2: Forest plot comparing each dose of the melatonin and the placebo regarding heart rate:

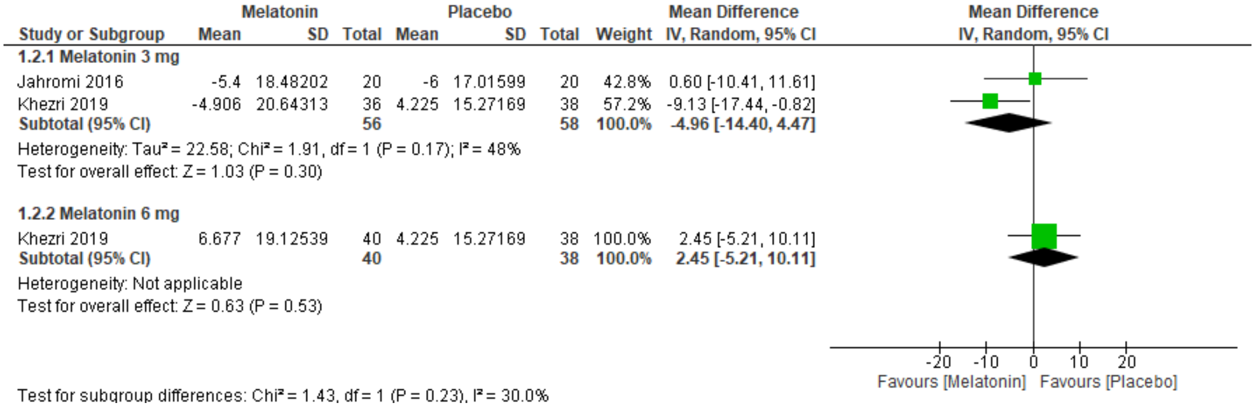

Figure S3: Forest plot comparing each dose of the melatonin and the placebo regarding mean arterial pressure:

	Melatonin			Placebo				Mean Difference
**Study or Subgroup**	**Mean**	**SD**	**Total**	**Mean**	**SD**	**Total**	**Weight**	**IV, Random, 95% CI**
**1.3.1 Melatonin 3 mg**								
Jahromi 2016	-12.4367	28.3024	20	-2.04667	27.24479	20	22.2%	-10.39 [-27.61, 6.83]
Khezri 2019	-19.859	17.21707	36	-19.978	16.92025	38	77.8%	0.12 [-7.66, 7.90]
**Subtotal (95% CI)**			**56**			**58**	**100.0%**	**-2.21 [-10.77, 6.35]**
Heterogeneity: Tau² = 8.75; Chi² = 1.19, df = 1 (P = 0.28); I² = 16%								
Test for overall effect: Z = 0.51 (P = 0.61)								
**1.3.2 Melatonin 6 mg**								
Khezri 2019	-20.336	16.82114	40	-19.978	16.92025	38	100.0%	-0.36 [-7.85, 7.13]
**Subtotal (95% CI)**			**40**			**38**	**100.0%**	**-0.36 [-7.85, 7.13]**
Heterogeneity: Not applicable								
Test for overall effect: Z = 0.09 (P = 0.93)								

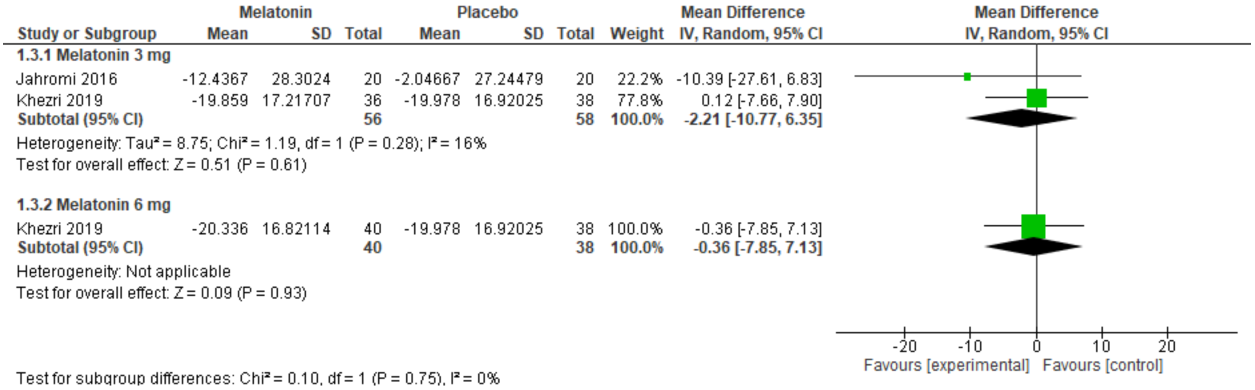

Test for subgroup differences: Chi² = 0.10, df = 1 (P = 0.75), I² = 0%

Figure S4: Forest plot comparing each dose of the melatonin and the placebo regarding the pain score:

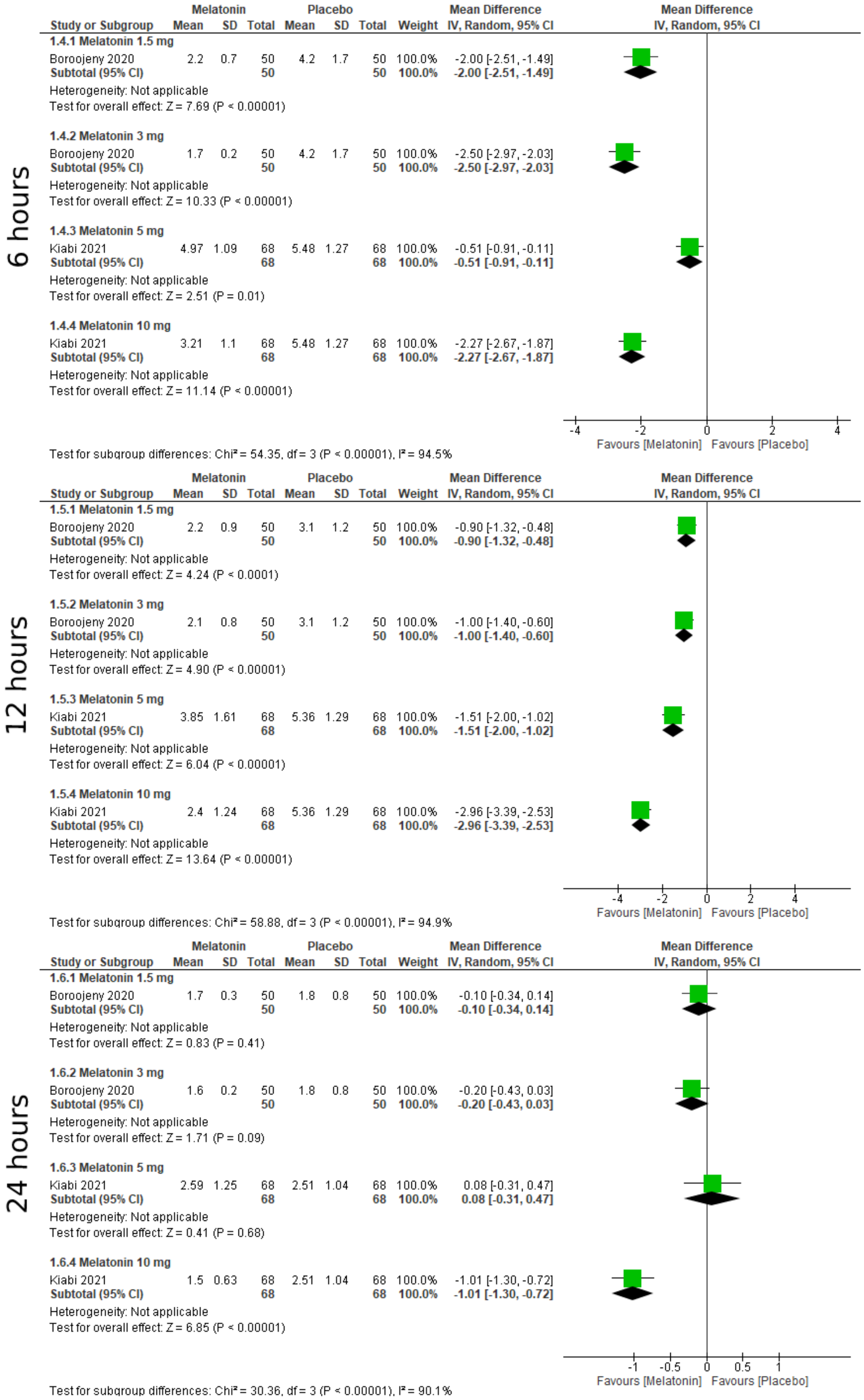

Figure S5: Forest plot comparing each dose of the melatonin and the placebo regarding the time to first analgesic request:

Study or Subgroup	Melatonin Mean	Melatonin SD	Melatonin Total	Placebo Mean	Placebo SD	Placebo Total	Weight	Mean Difference IV, Random, 95% CI
**1.7.1 Melatonin 1.5 mg**								
Boroojeny 2020	187.1	65.8	50	132.5	49.3	50	100.0%	54.60 [31.81, 77.39]
**Subtotal (95% CI)**			**50**			**50**	**100.0%**	**54.60 [31.81, 77.39]**
Heterogeneity: Not applicable								
Test for overall effect: Z = 4.70 (P < 0.00001)								
**1.7.2 Melatonin 3 mg**								
Boroojeny 2020	196.5	71.8	50	132.5	49.3	50	79.4%	64.00 [39.86, 88.14]
Khezri 2019	208.19	122.66	36	152.13	79.4	38	20.6%	56.06 [8.70, 103.42]
**Subtotal (95% CI)**			**86**			**88**	**100.0%**	**62.36 [40.85, 83.87]**
Heterogeneity: Tau² = 0.00; Chi² = 0.09, df = 1 (P = 0.77); I² = 0%								
Test for overall effect: Z = 5.68 (P < 0.00001)								
**1.7.3 Melatonin 5 mg**								
Kiabi 2021	172.2	123.6	68	121.8	103.8	68	100.0%	50.40 [12.04, 88.76]
**Subtotal (95% CI)**			**68**			**68**	**100.0%**	**50.40 [12.04, 88.76]**
Heterogeneity: Not applicable								
Test for overall effect: Z = 2.57 (P = 0.01)								
**1.7.4 Melatonin 6 mg**								
Khezri 2019	196.82	127.25	40	152.13	79.4	38	100.0%	44.69 [-2.13, 91.51]
**Subtotal (95% CI)**			**40**			**38**	**100.0%**	**44.69 [-2.13, 91.51]**
Heterogeneity: Not applicable								
Test for overall effect: Z = 1.87 (P = 0.06)								
**1.7.5 Melatonin 10 mg**								
Kiabi 2021	211.8	90.6	68	121.8	103.8	68	100.0%	90.00 [57.25, 122.75]
**Subtotal (95% CI)**			**68**			**68**	**100.0%**	**90.00 [57.25, 122.75]**
Heterogeneity: Not applicable								
Test for overall effect: Z = 5.39 (P < 0.00001)								

Test for subgroup differences: Chi² = 4.08, df = 4 (P = 0.39), I² = 2.1%

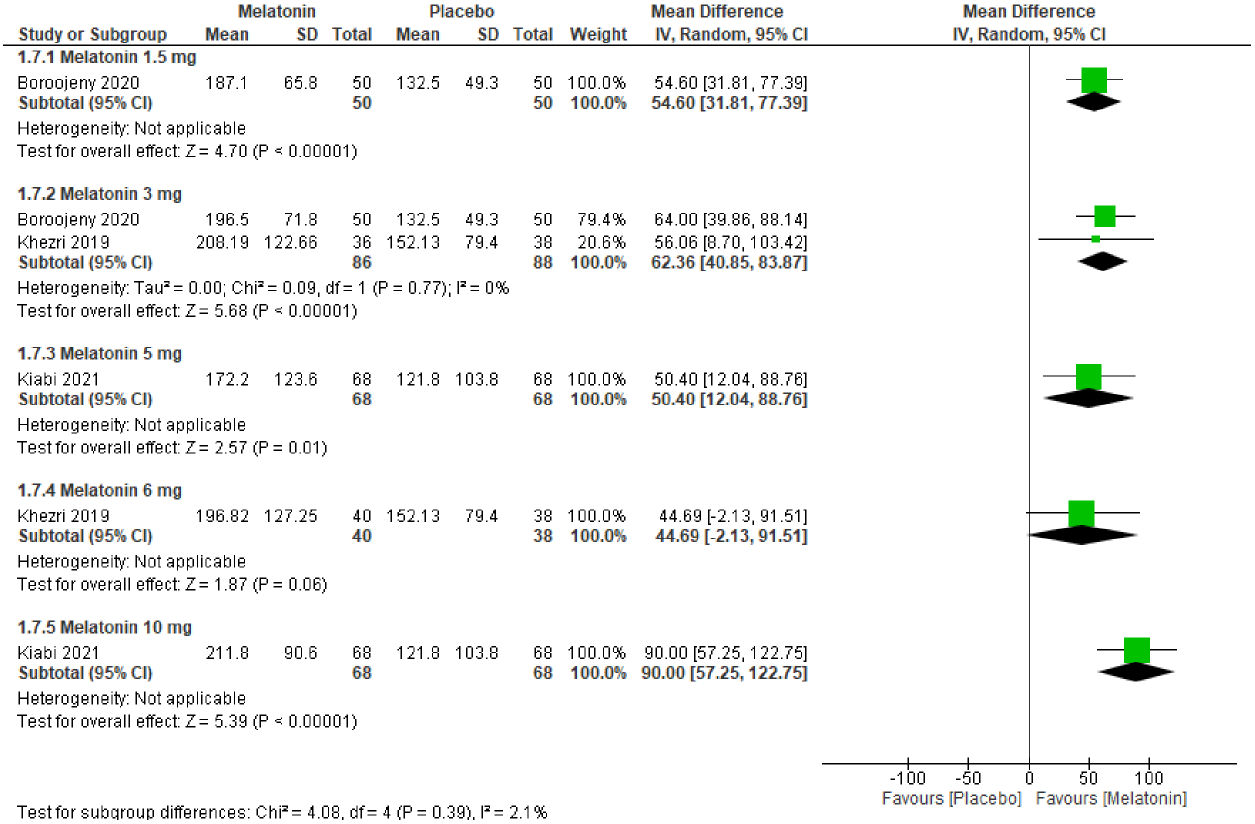

Figure S6: Forest plot comparing each dose of the melatonin and the placebo regarding the total blood loss:

Study or Subgroup	Melatonin Mean	Melatonin SD	Melatonin Total	Placebo Mean	Placebo SD	Placebo Total	Weight	Mean Difference IV, Random, 95% CI
**1.8.1 Melatonin 3 mg**								
Gowda 2022	1,440	170.31	30	1,357.17	162.82	30	52.2%	82.83 [-1.48, 167.14]
Khezri 2019	483.66	197.9557	36	470.86	193.2025	38	47.8%	12.80 [-76.39, 101.99]
**Subtotal (95% CI)**			**66**			**68**	**100.0%**	**49.39 [-19.17, 117.95]**
Heterogeneity: Tau² = 491.43; Chi² = 1.25, df = 1 (P = 0.26); I² = 20%								
Test for overall effect: Z = 1.41 (P = 0.16)								
**1.8.2 Melatonin 6 mg**								
Jayashree 2021	382	26.182	30	377	36.403	30	96.5%	5.00 [-11.05, 21.05]
Khezri 2019	467.05	187.2003	40	470.86	193.2025	38	3.5%	-3.81 [-88.30, 80.68]
**Subtotal (95% CI)**			**70**			**68**	**100.0%**	**4.69 [-11.07, 20.46]**
Heterogeneity: Tau² = 0.00; Chi² = 0.04, df = 1 (P = 0.84); I² = 0%								
Test for overall effect: Z = 0.58 (P = 0.56)								

Test for subgroup differences: Chi² = 1.55, df = 1 (P = 0.21), I² = 35.5%

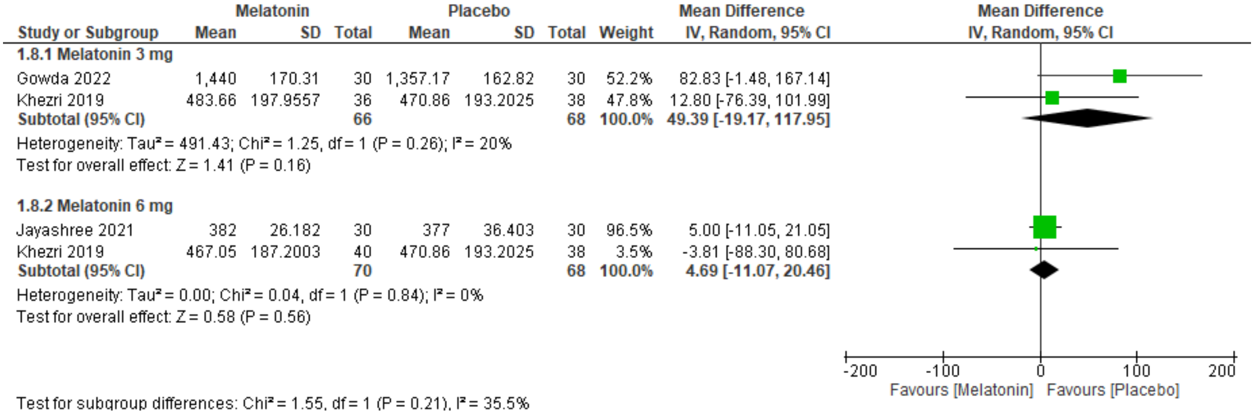

Figure S7: Forest plot comparing each dose of the melatonin and the placebo regarding the adverse events:

Nausea

Study or Subgroup	Melatonin Events	Melatonin Total	Placebo Events	Placebo Total	Weight	Risk Ratio M-H, Random, 95% CI
**1.9.1 Melatonine 1.5 mg**						
Boroojeny 2020	13	50	12	50	100.0%	1.08 [0.55, 2.14]
**Subtotal (95% CI)**		**50**		**50**	**100.0%**	**1.08 [0.55, 2.14]**
Total events	13		12			
Heterogeneity: Not applicable						
Test for overall effect: Z = 0.23 (P = 0.82)						
**1.9.2 Melatonine 3 mg**						
Boroojeny 2020	16	50	12	50	46.3%	1.33 [0.70, 2.52]
Khezri 2019	13	36	20	38	53.7%	0.69 [0.40, 1.16]
**Subtotal (95% CI)**		**86**		**88**	**100.0%**	**0.93 [0.49, 1.79]**
Total events	29		32			
Heterogeneity: Tau² = 0.13; Chi² = 2.50, df = 1 (P = 0.11); I² = 60%						
Test for overall effect: Z = 0.21 (P = 0.84)						
**1.9.3 Melatonine 6 mg**						
Khezri 2019	17	40	20	38	100.0%	0.81 [0.50, 1.29]
**Subtotal (95% CI)**		**40**		**38**	**100.0%**	**0.81 [0.50, 1.29]**
Total events	17		20			
Heterogeneity: Not applicable						
Test for overall effect: Z = 0.89 (P = 0.37)						

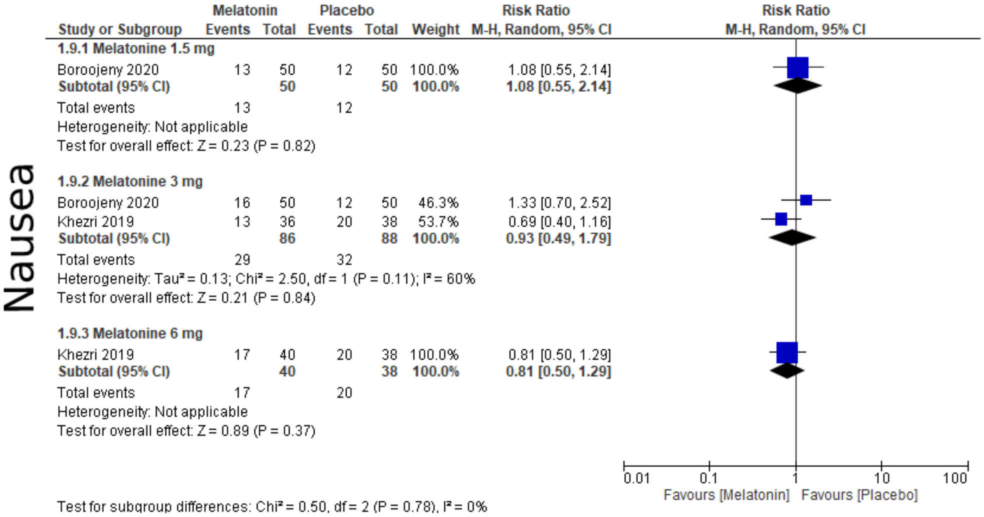

Test for subgroup differences: Chi² = 0.50, df = 2 (P = 0.78), I² = 0%

Vomiting

Study or Subgroup	Melatonin Events	Melatonin Total	Placebo Events	Placebo Total	Weight	Risk Ratio M-H, Random, 95% CI
**1.10.1 Melatonine 1.5 mg**						
Boroojeny 2020	8	50	5	50	100.0%	1.60 [0.56, 4.56]
**Subtotal (95% CI)**		**50**		**50**	**100.0%**	**1.60 [0.56, 4.56]**
Total events	8		5			
Heterogeneity: Not applicable						
Test for overall effect: Z = 0.88 (P = 0.38)						
**1.10.2 Melatonine 3 mg**						
Boroojeny 2020	7	50	5	50	47.3%	1.40 [0.48, 4.12]
Khezri 2019	5	36	12	38	52.7%	0.44 [0.17, 1.12]
**Subtotal (95% CI)**		**86**		**88**	**100.0%**	**0.76 [0.24, 2.36]**
Total events	12		17			
Heterogeneity: Tau² = 0.40; Chi² = 2.52, df = 1 (P = 0.11); I² = 60%						
Test for overall effect: Z = 0.47 (P = 0.64)						
**1.10.3 Melatonine 6 mg**						
Khezri 2019	12	40	12	38	100.0%	0.95 [0.49, 1.85]
**Subtotal (95% CI)**		**40**		**38**	**100.0%**	**0.95 [0.49, 1.85]**
Total events	12		12			
Heterogeneity: Not applicable						
Test for overall effect: Z = 0.15 (P = 0.88)						

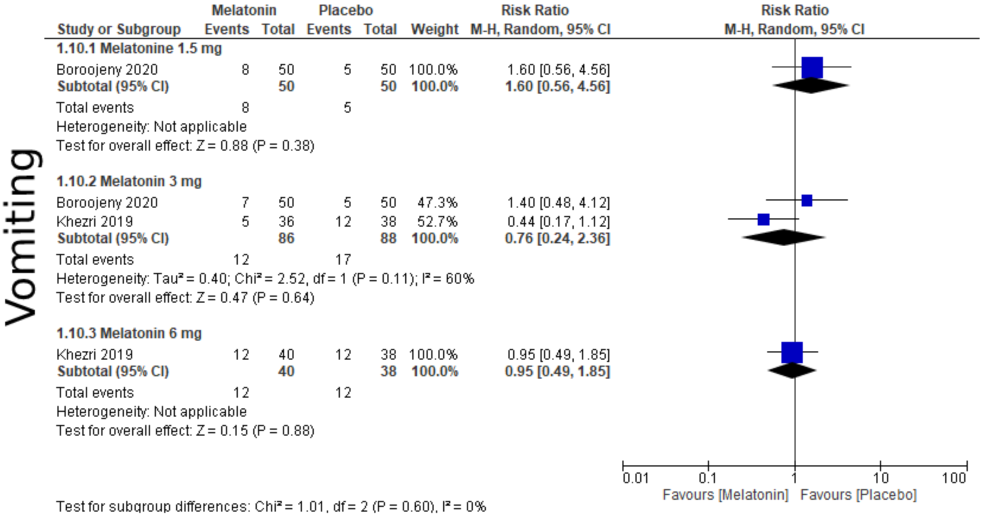

Test for subgroup differences: Chi² = 1.01, df = 2 (P = 0.60), I² = 0%

Headache

Study or Subgroup	Melatonin Events	Melatonin Total	Placebo Events	Placebo Total	Weight	Risk Ratio M-H, Random, 95% CI
**1.11.1 Melatonine 1.5 mg**						
Boroojeny 2020	11	50	9	50	100.0%	1.22 [0.56, 2.69]
**Subtotal (95% CI)**		**50**		**50**	**100.0%**	**1.22 [0.56, 2.69]**
Total events	11		9			
Heterogeneity: Not applicable						
Test for overall effect: Z = 0.50 (P = 0.62)						
**1.11.2 Melatonine 3 mg**						
Boroojeny 2020	7	50	9	50	76.5%	0.78 [0.31, 1.93]
Khezri 2019	2	36	4	38	23.5%	0.53 [0.10, 2.71]
**Subtotal (95% CI)**		**86**		**88**	**100.0%**	**0.71 [0.32, 1.57]**
Total events	9		13			
Heterogeneity: Tau² = 0.00; Chi² = 0.17, df = 1 (P = 0.68); I² = 0%						
Test for overall effect: Z = 0.85 (P = 0.40)						
**1.11.3 Melatonine 6 mg**						
Khezri 2019	18	40	4	38	100.0%	4.28 [1.59, 11.48]
**Subtotal (95% CI)**		**40**		**38**	**100.0%**	**4.28 [1.59, 11.48]**
Total events	18		4			
Heterogeneity: Not applicable						
Test for overall effect: Z = 2.88 (P = 0.004)						

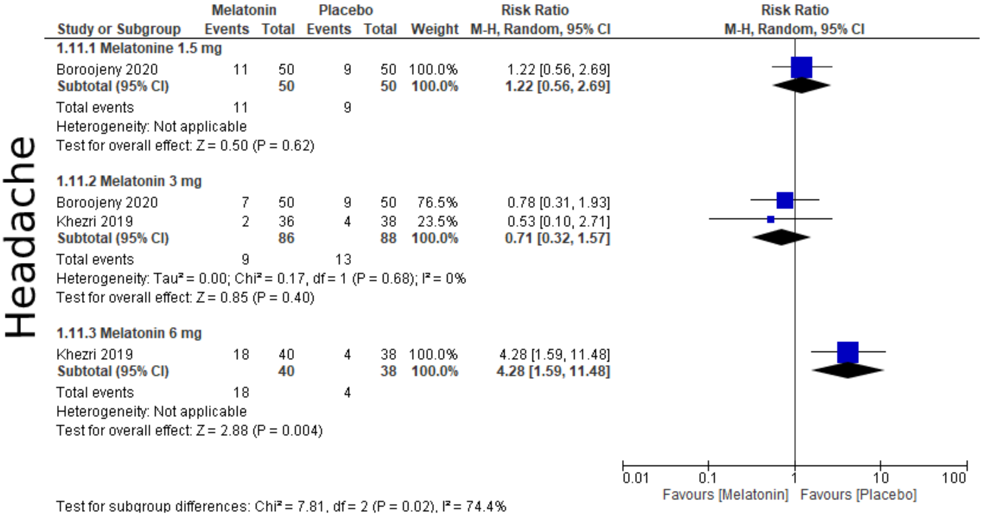

Test for subgroup differences: Chi² = 7.81, df = 2 (P = 0.02), I² = 74.4%
